# FGF9 induces functional differentiation to Schwann cells from human adipose derived stem cells

**DOI:** 10.7150/thno.38553

**Published:** 2020-02-03

**Authors:** Chia-Wei Huang, Shih-Yu Lu, Tzu-Chieh Huang, Bu-Miim Huang, H. Sunny Sun, Shang-Hsun Yang, Jih-Ing Chuang, Yuan-Yu Hsueh, Yi-Ting Wu, Chia-Ching Wu

**Affiliations:** 1 Institute of Basic Medical Sciences, National Cheng Kung University, Tainan 701, Taiwan; 2 Department of Cell Biology and Anatomy, National Cheng Kung University, Tainan 701, Taiwan; 3 Institute of molecular medicine, National Cheng Kung University, Tainan 701, Taiwan; 4 Department of Physiology, National Cheng Kung University, Tainan 701, Taiwan; 5 Division of Plastic Surgery, National Cheng Kung University Hospital, Tainan 704, Taiwan; 6 Institute of Clinical Medicine, National Cheng Kung University, Tainan 701, Taiwan; 7 International Center for Wound Repair and Regeneration, National Cheng Kung University, Tainan 701, Taiwan; 8 Department of Nursing, Tzu Hui Institute of Technology, Pingtung 926, Taiwan

**Keywords:** FGF9, FGFR2, adipose-derived stem cell, neurosphere, Schwann cell differentiation

## Abstract

**Rationale**: The formation of adipose-derived stem cells (ASCs) into spheres on a chitosan-coated microenvironment promoted ASCs differentiation into a mixed population of neural lineage-like cells (NLCs), but the underline mechanism is still unknown. Since the fibroblast growth factor 9 (FGF9) and fibroblast growth factor receptors (FGFRs) play as key regulators of neural cell fate during embryo development and stem cell differentiation, the current study aims to reveal the interplay of FGF9 and FGFRs for promoting peripheral nerve regeneration.

**Methods**: Different concentration of FGF9 peptide (10, 25, 50, 100 ng/mL) were added during NLCs induction (FGF9-NLCs). The FGFR expressions and potential signaling were studied by gene and protein expressions as well as knocking down by specific FGFR siRNA or commercial inhibitors. FGF9-NLCs were fluorescent labeled and applied into a nerve conduit upon the injured sciatic nerves of experimental rats.

**Results**: The FGFR2 and FGFR4 were significantly increased during NLCs induction. The FGF9 treated FGF9-NLCs spheres became smaller and changed into Schwann cells (SCs) which expressed S100β and GFAP. The specific silencing of FGFR2 diminished FGF9-induced Akt phosphorylation and inhibited the differentiation of SCs. Transplanted FGF9-NLCs participated in myelin sheath formation, enhanced axonal regrowth and promoted innervated muscle regeneration. The knockdown of FGFR2 in FGF9-NLCs led to the abolishment of nerve regeneration.

**Conclusions**: Our data therefore demonstrate the importance of FGF9 in the determination of SC fate via the FGF9-FGFR2-Akt pathway and reveal the therapeutic benefit of FGF9-NLCs.

## Introduction

Schwann cells (SCs), the principal myelinating glia in the peripheral nervous system (PNS), encompass axons to form a myelin sheath and to support neuronal function. Besides wrapping the axon to insulate and speed up the conduction of nerve impulses, the SCs also secrete critical growth factors and extracellular matrix to help maintain axons and neuronal survival. Upon peripheral nerve injury, axon demyelination and degradation occur in distal stumps during a process known as Wallerian degeneration 1. During this process, SCs participate in the clearance of debris and then dedifferentiate into repair SCs to form bands of Bungner in order to support and guide axonal regrowth via the upregulation of c-Jun signals 2. In cases involving an injured nerve, a nerve conduit is used to connect the two nerve stumps together; however, axonal regeneration in critical gap cases is still hindered by the lack of appropriate growth factors and guidance cues in the conduits 3. The transplantation of SCs can facilitate PNS recovery, but the shortage of autologous SCs represents a serious limitation for this clinical application 4.

Neural stem cells (NSCs) can differentiate into all neural lineage cells and can be used to replace, support, or guide nerve regeneration in both the central nervous system (CNS) and the PNS 5. There are several sources from which NSCs can be obtained, including isolation from the neonatal brain 6 or the differentiation of embryonic stem cells (ESCs), induced pluripotent stem cells (iPSCs) 7, bone marrow mesenchymal stem cells (BMMSCs) 8, adipose-derived stem cells (ASCs) 9 and placenta-derived multipotent cells 10. However, ethical deliberation is the greatest challenge facing the use of NSCs, except for adult or somatic stem cells. ASCs represent an ideal autologous cell source for nerve tissue regeneration 11. By transplanted into target tissue, ASCs can serve as a vector to deliver beneficial factors into damaged site for promoting regeneration 12. In addition, it can also reportedly undergo trans-differentiation into terminally differentiated neural lineage cells with the treatment of cytokines and growth factors 9. Culturing ASCs at high density in neural basal medium supplemented with B27 was previously shown to induce compact free-floating neurospheres with a similar genetic expression profile as NSCs 13. These neurospheres can further differentiate into neuronal, glial, and oligodendrocyte cells with the additional supplementation of fibroblast growth factor 2 (FGF2), brain-derived neurotrophic factor (BDNF) and epidermal growth factor (EGF) 14. Our laboratory previously developed a convenient method with which to induce the formation of neurospheres by seeding ASCs onto a chitosan-coated surface 15. These neurospheres were composited by a mixed population of neural lineage-like cells (NLCs) with the inner sphere showing positive neuronal cells for Nestin and neuralfilament heavy chain (NFH); the outer surface exhibited glial fibrillary acidic protein (GFAP)-positive glial cells. The application of ASC-derived NLCs into a chitosan-coated nerve conduit further demonstrated superior regeneration of the PNS and the repair of sciatic nerve injury in rats 16. We also discovered that injecting these NLCs into a chitosan-coated conduit could modulate injury-induced inflammatory responses via the 5-lipoxygenase (5-LO) pathway.

The mammalian FGF family contains 18 ligands and 4 transmembrane FGF receptors (FGFRs) 17. FGF is cellular context-dependence with highly complex signal transduction pathways for various biological processes as well as neural development and neuroprotection 18. Several FGFs regulate neural development during embryogenesis and can be used to manipulate the fate decision of stem cells into specific neuronal or glial cell types 19. The cocktail of FGF2 or FGF8 can induce neuron differentiation in ESCs and human iPSCs 20-22. Combination of several critical factors, including FGFs, Sonic hedgehog (SHH), or retinoic acid (RA), guided the neuronal induction in human iPSCs 23, 24. Among them, the FGF2 is an essential factor with which to supplement the culture of human iPSCs from the naïve stage 25. FGF9, also known as glia-activating factor, is initially isolated from the culture medium of a human glioma cell line that can modulate the development and survival of neurons and glia in the CNS 26. FGF9/16/20 is highly expressed in the 16 cell-embryo to drive neural tissue formation 27. Aberrant FGF9 expression is associated with many human diseases, especially in the nervous system, including Alzheimer disease, Parkinson's disease and Huntington's disease 28-30. FGF9 also exerts neuroprotective effects against oxidative insults by activating Akt and ERK signaling 31. However, the FGF9 is up regulated in the demyelinating lesions to inhibit the myelination and secrete the pro-inflammation chemokines by astrocytes in multiple sclerosis patients 32. Due to the complexity of FGF-FGFR ligand-receptor interactions, the molecular mechanisms underlying FGF-mediated stem cell fate determination for adult stem cell are poorly understood. Treating the MSC monolayer culture with supplementary of FGF2 induced the FGFR1 and FGFR4 gene expressions whereas inhibited the expression of FGFR2 and FGFR3 33. FGF9 has a higher affinity for FGFR2 and FGFR3, especially the C form 34. Although the FGF signaling are highly involved in neural differentiations, the FGF-FGFR interactions for ASCs and FGFR profile changes during NLCs induction are still unknown.

The current study aimed to investigate the FGFR profile in ASCs during sphere formation. Since SCs play important roles for peripheral glia, we also interested to identify the glia-related role of FGF9 in the differentiation of NLCs and its therapeutic potential for peripheral nerve injury. We hypothesized that the FGFRs on NLCs during sphere formation are different from ASCs under adhesive culture and that the FGF9 ligand may pivot the cell fate in neural lineages. The discovery of current research can help us to understand the molecular interactions of chitosan-coated surface with presentation of FGF9 during stem cell differentiation, especially for the potential roles and signaling pathway for Schwann cells. We further used the rat sciatic injury and nerve conduit to investigate the repair and regeneration potential of these differentiated cells with or without the treatment of FGF9. The results of current study revealed the role of FGF9 to guide the differentiation of ASCs towards Schwann cells to promote the formation of myelin sheath and nerve regeneration.

## Results

### Changes in the FGFR profile during the sphere formation of ASCs

**Figure 1:**
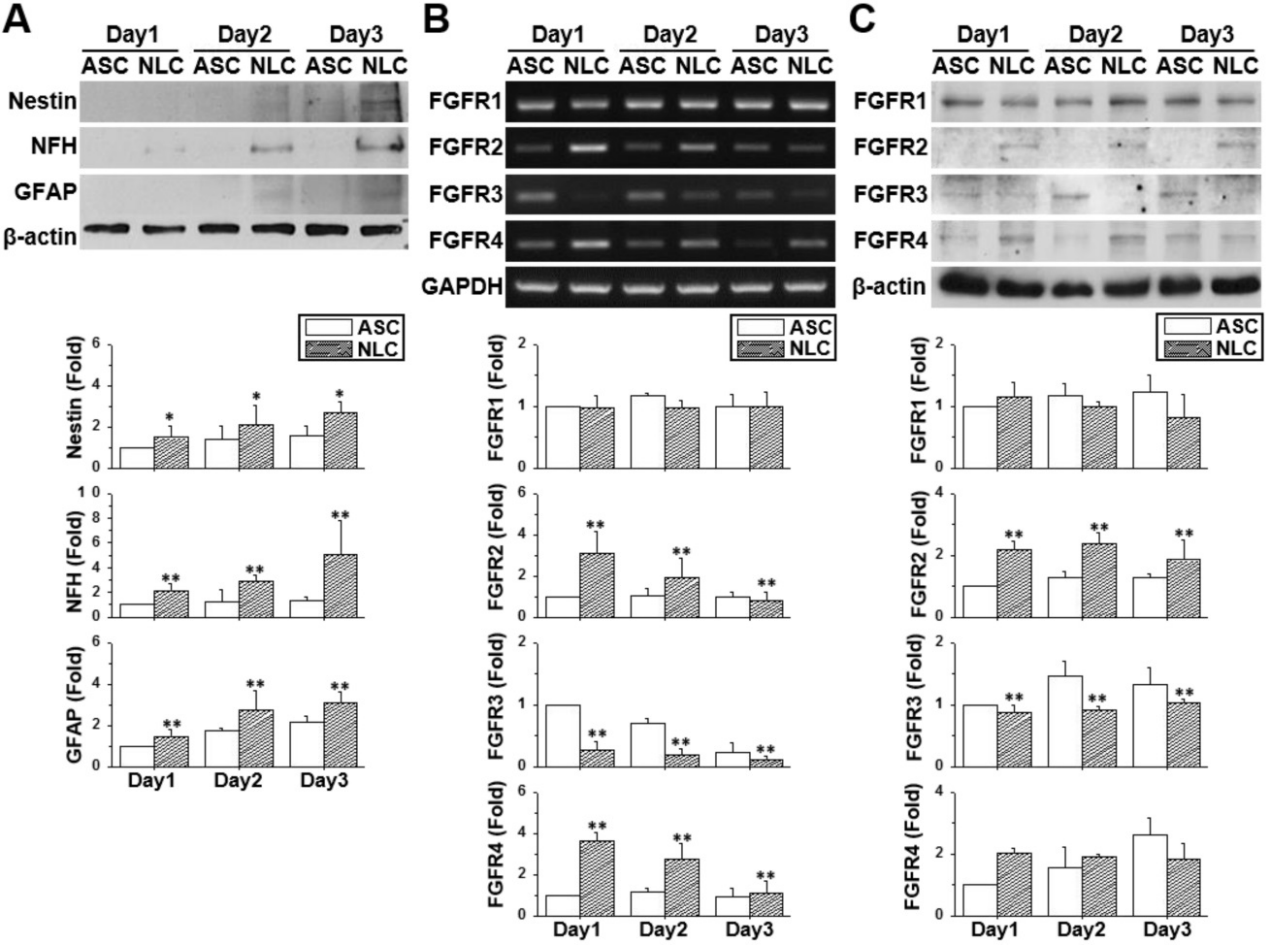
** The profile of neural markers and FGFRs changed during neural induction.** ASCs were seeded onto a chitosan-coated dish for neural induction and cells harvested between day 1 and day 3. (A) The increased protein expression using western blotting for Nestin, NFH, and GFAP demonstrated the successful induction of neural lineage-like cells (NLCs) from ASCs. Both RT-PCR (B) and western blotting (C) showed the upregulation of FGFR2 and FGFR4 during the induction of NLCs. FGFR3 was downregulated in NLCs. Data represent means ± SD of three independent biological repeats. Data were normalized against undifferentiated ASCs. *p < 0.05, **p < 0.01 compared to undifferentiated ASCs.

The neural-like induction of ASCs was accomplished by culturing ASCs on a chitosan-coated dish for three days. The ASCs gradually aggregated together and formed a dense spherical structure showing gradual increases in the protein (Figure 1A) (p < 0.05) and mRNA expression (Figure S1) of neural differentiation markers. The increased expression of the neural markers, Nestin, NFH, NeuN and the GFAP gene was indicative of a mixed population of NLCs, including neural progenitor cells, neuronal cells and glial cells. The expression patterns of FGFRs were measured by using reverse transcribed polymerase chain reaction (RT-PCR) (Figure 1B) and western blotting (Figure 1C) on different days of cell culture or sphere formation. Both the mRNA and protein expression levels of FGFR1 remained unchanged in ASCs and NLCs, although levels of FGFR2 and FGFR4 were increased during the induction of NLCs (p < 0.01). On the contrast, levels of FGFR3 were reduced in NLCs on day 1 and stayed at low levels during induction (p < 0.01). The existence of a different FGFR profile between ASCs and NLCs indicated the involvement of FGF-FGFR signaling in the neural differentiation of ASCs. In addition, the unchanged levels of FGFR1 suggested only a minor involvement, or less sensitivity to relative ligands, such as FGF2, during the differentiation of ASCs differentiation using spheroid formation.

### FGF9 guided the differentiation of spheres towards a Schwann cell lineage

**Figure 2:**
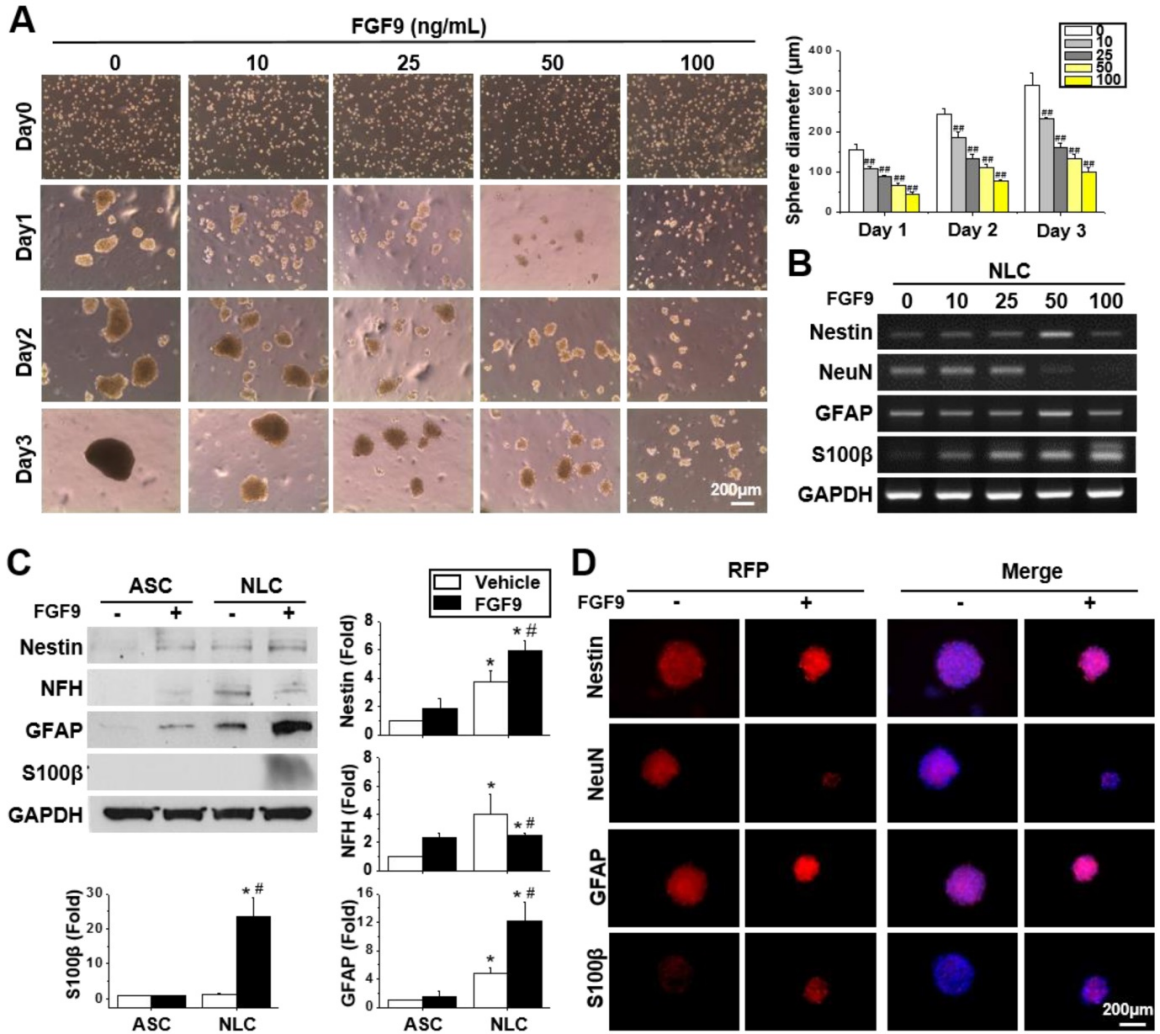
** FGF9 directed the differentiation of neurospheres into a Schwann cell (SCs) lineage. (**A) Different concentrations of FGF9 were added into the culture medium during NLCs induction. The gross morphology of spheres revealed that the size of neurospheres was reduced as the concentration of FGF9 concentration increased (0 to 100 ng/ml). ##p < 0.01 compared to vehicle-treated NLCs. (B) The RT-PCR results showed the gene expression of Nestin, GFAP, and S100β increased with FGF9 treatment, while that of NeuN decreased; these changes occurred in a dose-dependent manner. With the administration of FGF9 (50ng/ml), the induction of GFAP and S100β protein expression in SCs was observed by western blotting (C) and immunofluorescent staining (D) after 3 days of induction. The protein expression level was illustrated by staining of specific antibodies followed by RFP labeling. The cell number and sphere size were illustrated by DAPI (blue color in merged images). Scale bar in phase image: 200 μm. Scale bar in fluorescent image: 200 μm. Data represent the mean ± SD of three independent biological repeats. *p < 0.05 compared to undifferentiated ASCs. #p < 0.05 compared to vehicle-treated NLCs.

Different concentrations of FGF9 were supplemented into the culture medium when seeding ASCs on a chitosan-coated dish. Instead of forming a single large sphere on Day 3 of NLC induction, the addition of FGF9 caused ASCs to form smaller spheres in a dose-dependent manner (Figure 2A) (p < 0.01). A switching of cell fate to a different neural lineage was discovered when adding different concentrations of FGF9 during sphere formation (Figure 2B). With increasing levels of FGF9, the expression of the neuronal marker, NeuN, decreased, whereas the expression of the glial lineage markers, GFAP and particularly, S100β, was augmented in NLCs. The addition of FGF9 increased the expression of Nestin suggesting that the remaining FGF9-induced NLCs showed neural progenitor properties. The protein expression of these neural lineage markers were confirmed by western blotting (Figure 2C) and immunofluorescent staining (Figure 2D) when treating with FGF9 (50 ng/ml) after the induction of NLCs for 3 days. The increased levels of S100β suggested the fate determination of NLCs towards SCs under FGF9 treatment (p < 0.05). The early makers of SCs were also confirmed by Sox10 and Oct6 inductions after formation of NLCs (Figure S2). The administration of FGF9 to NLCs further increased both of Sox10 and Oct6 expressions.

FGF9 was downregulated when seeding ASCs on a chitosan-coated surface to form NLC spheres (Figure S3A) (p < 0.05). Thus, the autocrine effect of FGF9 may less influence in NLCs as comparing to the ASCs. Since FGF9 was previously reported to promote osteogenesis in BMMSCs 35, we tested whether the addition of FGF9 would alter the expression of the osteogenesis gene in ASCs and NLCs. No significant change was detected for osteogenesis or chondrogenesis in the mRNA expression of aggrecan, collagen type 2, and SOX9, in both ASCs and NLCs treated with different concentrations of FGF9 (Figure S3B). The BrdU assay was performed to investigate the cell proliferation effect of FGF9 in neurospheres. Adherent ASCs showed BrdU-positive proliferating cells, while most of the cells in NLCs became quiescent after sphere formation and did not proliferate in response to the addition of FGF9 (Figure S3C). Therefore, rather than FGF9 promoting cell proliferation, the predominant role of FGF9 treatment in the induction of NLCs appears to be the regulation of cell fate for SC differentiation.

### FGF9 transiently phosphorylated Akt in NLCs and promoted the differentiation of SCs via the FGFR2-Akt axis

**Figure 3:**
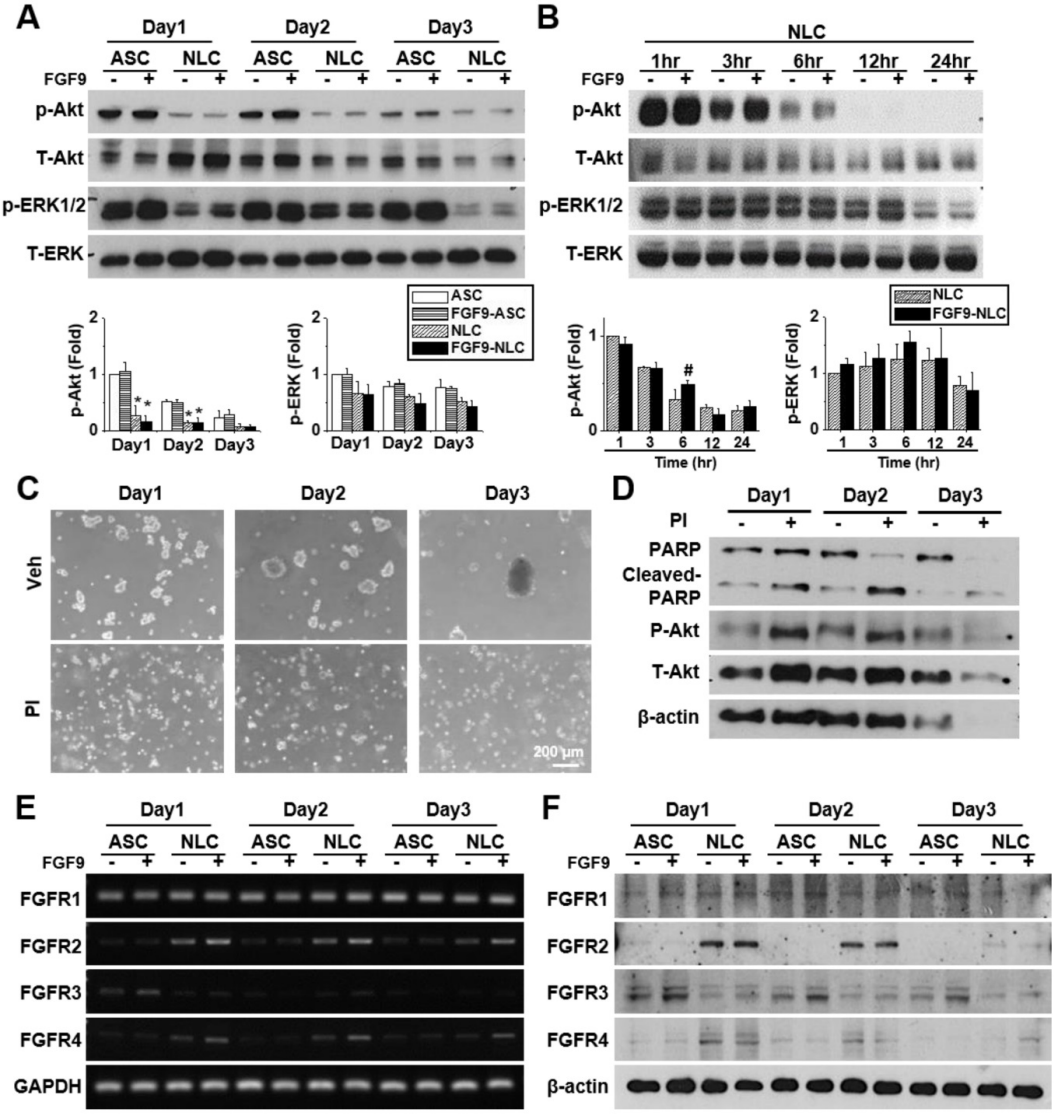
** FGF9 treatment activates FGFR2 and Akt signaling during the induction of NLCs.** (A) ASCs and NLCs were treated with or without FGF9 (50ng/ml) at the indicated time points to investigate the phosphorylation of Akt and ERK downstream signaling. The dephosphorylation of Akt and ERK were observed from the first day of NLC induction. (B) Although Akt and ERK were continuously dephosphorylated within 24hr of NLCs induction, the FGF9 treatment induced a transient activation of Akt at 6 hr. (C) During the induction of NLCs, the administration of phosphatase inhibitor (PI) abolished sphere formation. (D) Blocking Akt dephosphorylation and preventing sphere formation by using PI treatment increased PARP cleavage (Cleaved-PARP) for cell death. Although FGF9 treatment slightly induced the gene expression of FGFR2 and FGFR4 (E), the protein expression level remained unchanged regardless of FGF9 treatment (F). p-Akt, phosphorylated Akt; T-Akt, total Akt; p-ERK, phosphorylated ERK; T-ERK, total ERK. Scale bar in phase image: 200 μm. Data represent the mean ± SD of three independent biological repeats. For A, *p < 0.05 compared to undifferentiated ASCs. For B, #p < 0.05 compared to vehicle-treated NLCs.

FGFs play a vital role in cellular function by binding to FGFRs and activating downstream signaling pathways. FGF9 is known to activate Akt and MAPK phosphorylation in many cells. Here, we investigated the effects of FGF9 on Akt, ERK, and JNK phosphorylation in both ASCs and NLCs at different culture times. We observed the de-phosphorylation of Akt and ERK from day 1 to day 3 in NLCs during sphere formation as comparing to ASCs (Figure 3A, *p < 0.05). JNK phosphorylation showed little difference when compared between ASCs and NLCs (Figure S4A). To further investigate de-phosphorylation patterns over shorter time points, NLCs with or without FGF9 treatment, were additionally measured at 1, 3, 6, 12, and 24 hr after seeding on a chitosan-coated surface (Figure 3B and Figure S4B). FGF9 caused a transient increase in p-Akt at 6 hr (#p < 0.05), whereas the continuous de-phosphorylation of Akt was observed within 24 hr of sphere formation (Figure 3B). In order to perturb this de-phosphorylation, we used a phosphatase inhibitor (PI, 1:1000, Merck) during sphere formation; this prevented ASCs from forming neurospheres (Figure 3C). The de-phosphorylation of p-Akt was prevented under PI treatment; however, these suspending ASCs underwent apoptosis with occurrence of cell debris and an increase in the expression of cleaved-PARP protein after 3 days of induction (Figure 3D). The administration of FGF9 increased the mRNA expression of FGFR2 and FGFR4 (Figure 3E, quantification in Figure S4C) (p < 0.05); however, there was no considerable upregulation in protein levels (Figure 3F, quantification in Figure S4D). This might be due to FGF9 increasing the transcriptional activity of FGFR2 and FGFR4, although protein synthesis was blocked during post-transcriptional regulation. Although the FGFR2 and FGFR4 in western blotting still showed higher expression in NLCs when comparing with ASCs, we observed a gradually decrease of FGFR2 and FGFR4 in NLC between day 2 and day 3 as comparing to Day 1. The essential rapid Akt de-phosphorylation during sphere formation suggested an important role of phosphatase or protein degradation during the induction of NLCs. The transient Akt phosphorylation prior to the de-phosphorylation may provide some important signaling aspects for determining cell fate.

**Figure 4:**
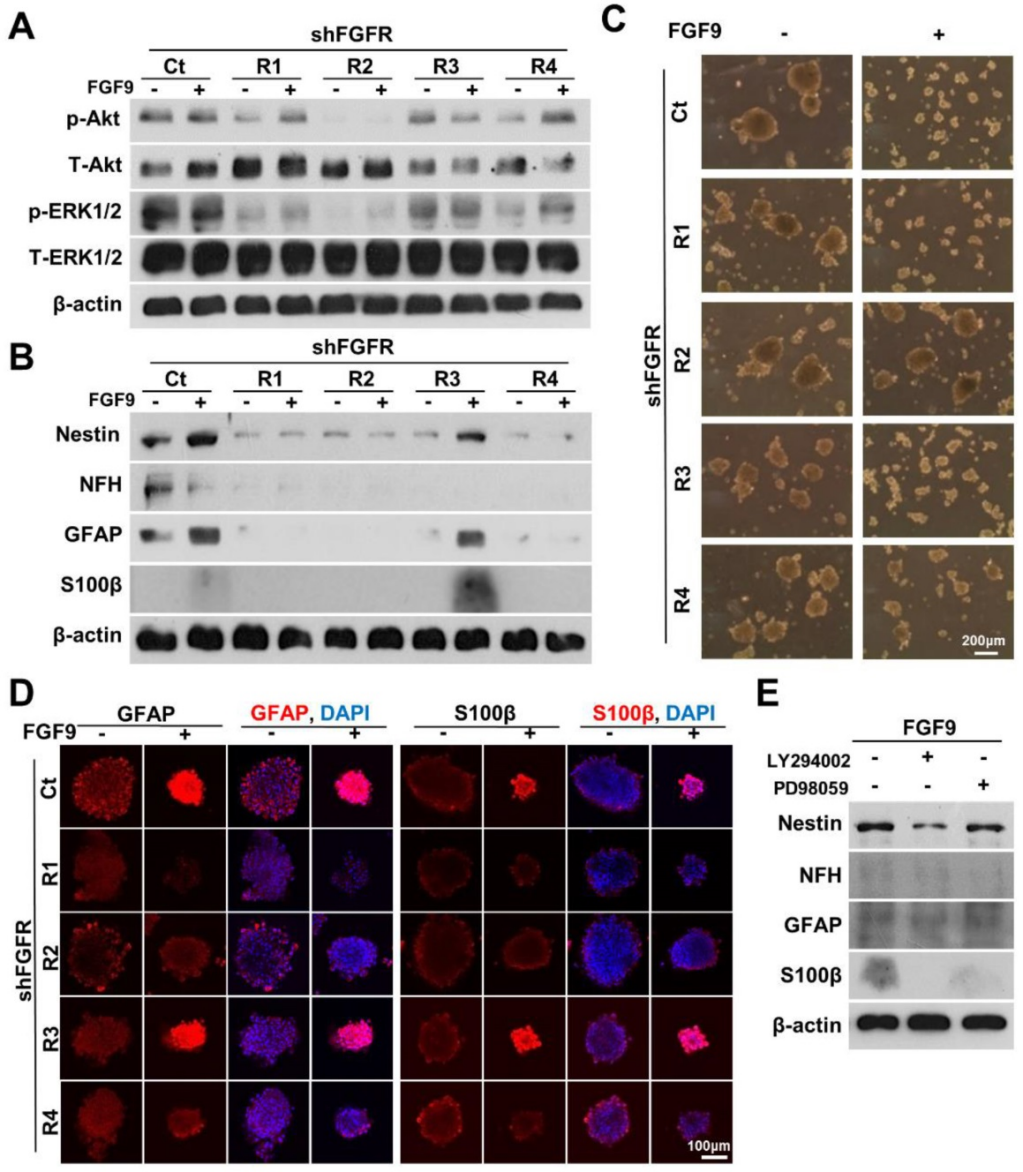
** Blocking FGFR2-Akt signals abolished FGF9-induced SC differentiation.** (A) shFGFR2 abolished FGF9-induced Akt and ERK phosphorylation. (B) Blocking FGFR2 prohibited FGF9-induced SC differentiation by reducing the expression of GFAP and S100β. NLC induction was also inhibited by shFGFR1 and shFGFR4, but not by shFGFR3. (C) Phase images showing that the reduction of sphere size by FGF9 treatment was hindered by shFGFR2. (D) Immunofluorescent staining of GFAP and S100β further confirmed the abolishment of SC markers and sphere size when knocking down FGFR2. (E) Inhibition of Akt phosphorylation by LY294006 (10μM) reduced the levels of Nestin, NFH, GFAP, and S100β protein expression and abolished FGF9- induced SC differentiation in NLCs. Application of the ERK inhibitor PD98059 (10μM) also led to a reduction in the expression of S100β, but not Nestin and GFAP. N=3. Scale bar in phase image: 200 μm. Scale bar in fluorescent image: 100 μm.

To identify the main receptor for FGF9 in facilitating the differentiation of NLCs into SCs, we specifically knocked down each FGFR by transfecting FGFR1, FGFR2, FGFR3, and FGFR4 shRNA before forming neurospheres. The specificity and efficiency of each shFGFR were also measured to confirm that the knockdown of these target FGFR proteins was efficient (Figure S5A). ASCs were transfected with specific shFGFRs one day prior to NLC induction and FGF9 application. Only the knockdown of FGFR2 blocked the Akt signals during NLCs induction (Figure 4A). Silencing FGFR2 (shFGFR2) abolished the expression of the neural protein markers, Nestin, NFH, GFAP, and S100β in both NLCs and FGF9-induced NLCs (FGF9-NLCs) after 3 days of induction (Figure 4B). In addition to shFGFR2, shFGFR1 and shFGFR4 also showed similar outcomes and significantly reduced the neural differentiation (Figure 4B, quantification in Figure S5B) (p < 0.05). The reduction of sphere size in FGF9-NLCs was reversed after knocking down FGFR2, but not with the other shFGFRs (Figure 4C). The perturbations of shFGFR upon expression of the glia makers, GFAP and S100β, were confirmed by taking confocal images of NLCs and FGF9-NLCs (Figure 4D). The distribution of GFAP-positive cells on the peripheral surface of NLC spheres became homogeneously expressed in smaller FGF9-NLCs spheres (Figure 4D, GFAP staining Ct spheres with or without FGF9). The shFGFR2 spheres were larger in size than in the Ct spheres of FGF9-NLCs along with the disappearance of GFAP and S100β expression after FGFR2 silencing (Figure 4D). Although the knockdown of FGFR1 and FGFR4 did not change the size of the spheres, the induction of glia (Figure 4D), and other neural makers (Figure S6), was significantly inhibited (Figure S5B, p < 0.05), even in the presence of FGF9. These results indicate the important roles of FGFR1, FGFR2, and FGFR4 in the neural differentiation of ASCs. Of these, FGFR2 plays a major role in FGF9-induced Akt phosphorylation and its subsequent SC differentiation. To further confirm the essential role of FGF9-induced transient Akt phosphorylation in SC fate commitment, we added specific inhibitors of Akt (LY294002) or ERK (PD98059) phosphorylation inhibitors into the FGF9-containing medium during the induction of NLCs (Figure 4E). The expression of Nestin, NFH and GFAP protein, and in particular, S100β, were reduced in FGF9-NLCs following LY294002 treatment. PD98059 also resulted in minor interference of FGF9-induced glial differentiation in NLCs, but this difference was not statistically significant (especially for GFAP expression). Collectively, these data demonstrated that SC differentiation into NLCs under FGF9 was orchestrated by the FGFR2-Akt signaling pathway.

### FGF9-induced NLCs participated in the re-myelination of injured sciatic nerve

**Figure 5:**
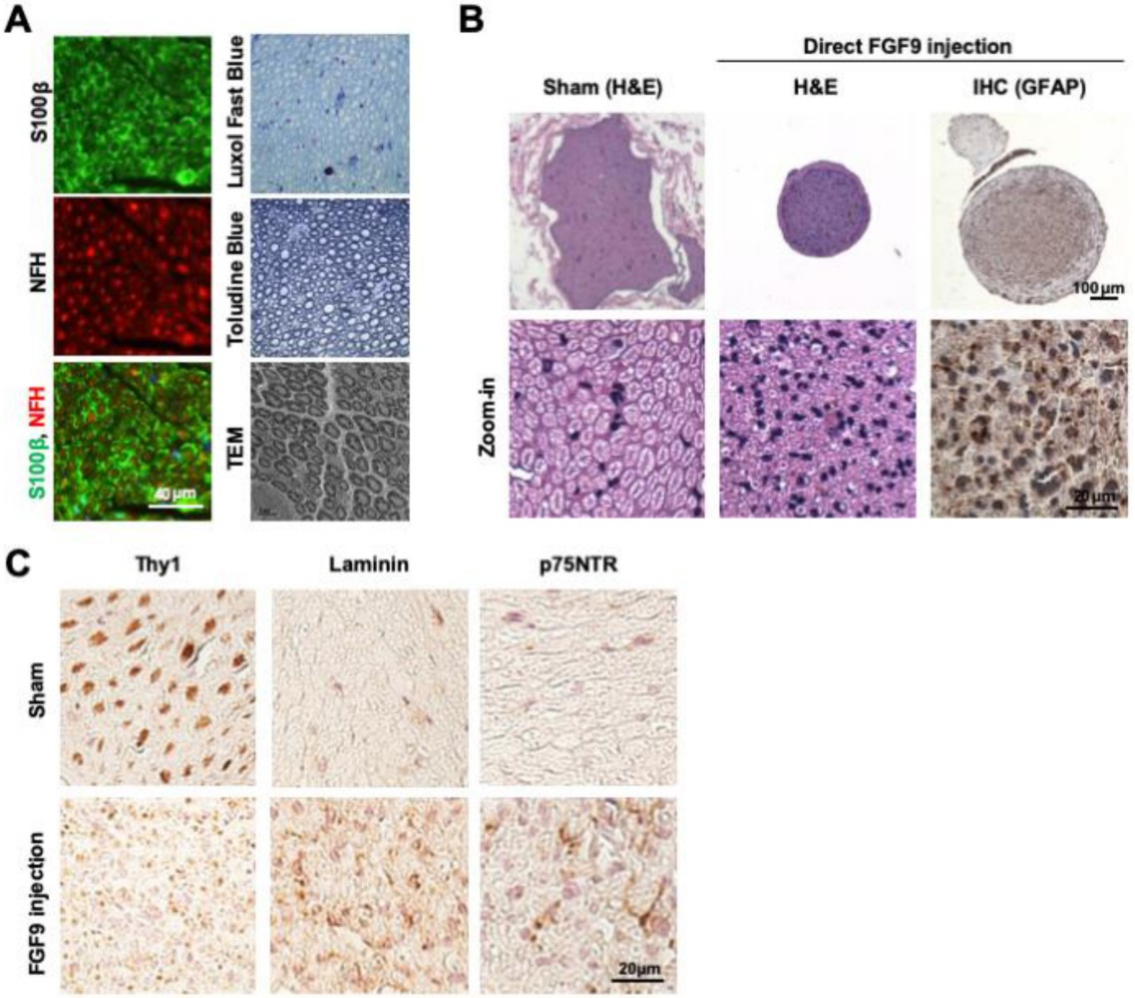
** Direct FGF9 administration into the nerve conduit caused a deterioration in nerve regeneration in a sciatic nerve transection model.** (A) The structure of intact sciatic nerve was revealed by the immunofluorescent staining of neural filament heavy-chain (NFH) for axons and S100β for the surrounding myelin sheath. The structure of the myelin sheath was further visualized by Luxol Fast Blue staining on paraffin sections, Toludine Blue staining on semi-thin sections, and by transmission electron microscopy (TEM). (B) The nerve transection model was created in an adult SD rat and then bridged with a chitosan-coated conduit (CC) for drug or cell application. Direct FGF9 administration (50 ng/mL) into the conduit caused severe fibrotic scar formation as shown by H&E and immunohistochemical (IHC) GFAP staining; this prevented nerve regeneration after 6 weeks of injury. (C) The fibrotic scar was confirmed by decreased Thy1 staining and increased laminin and p75NTR in direct FGF9 administration group by utilizing IHC staining.

Cross-sections of intact sciatic nerve revealed the beautiful architecture of axon-myelin bundles. We demonstrated that the axons (NFH staining, red) were surrounded by myelin sheath which was positively stained by S100β (S100β staining, green) (Figure 5A). The staining of myelin by Luxol Fast Blue and Toluidine Blue demonstrated that the myelin sheath was intact in sections of healthy nerve. The ultrastructure of the myelin sheath was also observed by transmission electron microscopy (TEM) (Figure 5A). To test whether FGF9 could promote nerve regeneration, we directly applied 50 ng/ml of FGF9 into the nerve conduit after transection injury to the sciatic nerve in rats. However, the direct administration of FGF9 triggered the formation of fibroblastic-like tissue (Figure 5B, H&E staining). In comparison to our previous results of nerve conduit without FGF9 16, we also observed the potential of induce the fibrotic glial scar morphology (Figure 5B, GFAP IHC staining) after injury which hindered axonal regrowth. The fibrotic scar formation in the nerve tissue after direct FGF9 administration was also confirmed by decreasing of IHC staining for Thy1 and increasing of both Laminin and p75NTR staining (Figure 5C).

**Figure 6:**
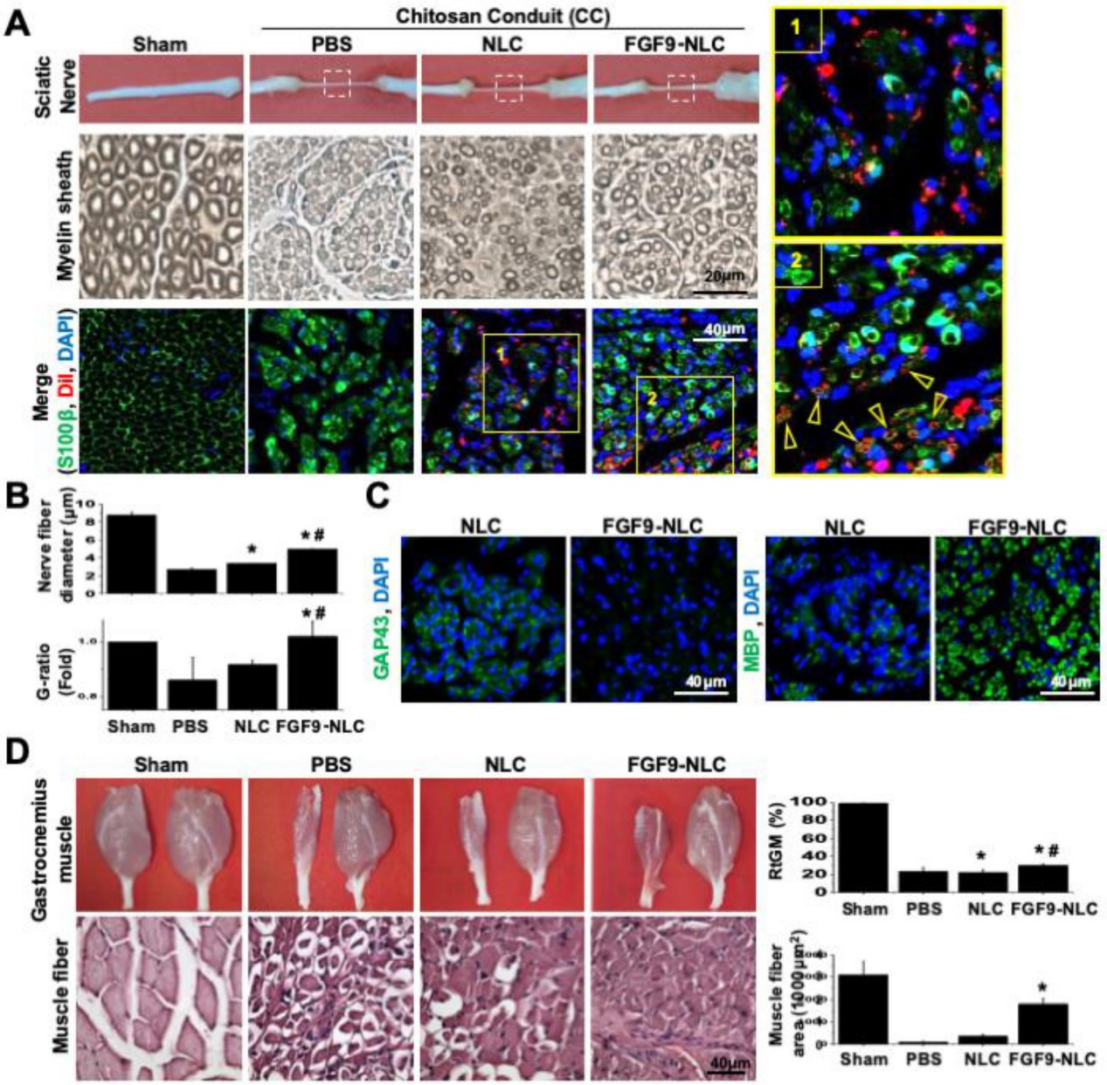
** Application of FGF9-induced NLCs promoted myelin sheath formation and regenerated injured nerve.** (A) NLCs or FGF9-induced NLCs (NLC-FGF9) were applied into the nerve conduit (CC) to bridge the transected nerves. Images of gross morphology (1^st^ row) show the regenerated sciatic nerve after 6 weeks of injury. P: proximal nerve; D: distal nerve. Myelin structure across different treatments was revealed by semi-thin sections (2^nd^ row) in the middle section of nerve tissue (white box region of 1^st^ row). The immunofluorescent staining of S100β visualized the co-localization of transplanted Dil-labeled FGF9-NLCs on the circular myelin sheath structure (arrows). Zoom-in image of the yellow box for NLC (area 1) and FGF9-NLCs (area2) treatments. (B) Quantitative analysis showed improvements in nerve fiber diameter and G-ratio of myelin sheath following FGF9-NLCs treatment. (C) Immunofluorescent staining of GAP43 revealed immature SCs on myelination in NLC treatment, whereas the application of FGF9-NLCs led to positive staining with the mature SC marker MBP. (D) The recovery of the sciatic nerve was illustrated by the obvious prevention of muscle atrophy in the gastrocnemius muscle. Quantitative results of RGMW and gastrocnemius muscle fiber cross section area. Scale bar: 40 μm. N=3. Data are represented as the mean ± SD of three independent biological repeats. *p < 0.05 compared to PBS. #p < 0.05 compared to NLC.

Since the *in vitro* application of FGF9 to NLCs led to the differentiation of SCs, we further investigated the therapeutic potential of cell-based therapy by applying NLC- or SC-fate committed FGF9-NLCs into the nerve conduit. After NLC induction, the spheres were rinsed and re-suspended to separate cells; cells were then labelled with DiI (red fluorescent dye) for cell tracing. Six weeks after injury, the nerve tissues were harvested for histological evaluations. The gross morphology showed that the nerve receiving an injection of FGF9-NLCs had a larger diameter of regenerated nerve (Figure 6A, 1^st^ row of gross pictures). Semi-thin sectioning showed that the application of FGF9-NLCs increased myelin sheath and sciatic nerve regeneration (Figure 6A, 2^nd^ row for myelin sheath). Quantifying the myelin structure, it was clear that the administration of FGF9-NLCs significantly increased the diameter of regenerating nerves and the G-ratio of myelin sheath as compared to phosphate-buffered saline (PBS) and NLCs treatment (Figure 6B) (p < 0.05). The myelin sheath area was also calculated and confirmed the increases of myelination with FGF9-NLCs treatment (Figure S7A). The specific roles played by the injected cells were further illustrated by tracing DiI-labeled cells (Figure S7B) with the immunofluorescent staining of S100β (Figure 6A, 3^rd^ row for immunofluorescent staining). In addition, the IF staining of laminin showed the fibrotic scar in PBS group. On the other hand, the formation of fibrotic scar was inhibited in both NLCs and FGF9-NLCs transplanted groups (Figure S7C). The mature myelin sheath structure was revealed by S100β staining in Sham-operated nerve. The injured nerves showed high levels of S100β staining, but did not show circular myelin sheath morphology, thus indicating the presence of immature SCs in PBS treatment (Figure 6A, 3^rd^ row of PBS group). The NLCs without FGF9 treatment (DiI-labeled NLCs) stayed close to the re-growing axons, but did not co-localize with S100β staining (Figure 6A, 3^rd^ row of NLCs group and zoom-in image of area 1). Since the application of NLCs also promoted nerve regeneration (as shown by our current data and our previously published results 16), the beneficial outcome might occur through paracrine secretions from neighboring DiI-labeled NLCs. In contrast, the co-localization of S100β expression on the circular myelin sheath and DiI-labeled cells suggested that the FGF9-NLCs differentiated into Schwann cells and directly participated in the re-myelination of regenerated myelin sheath (Figure 6A, 3^rd^ row of FGF9-NLCs group and arrows in area 2 image). Staining with a marker of immature SCs, GAP43, we found that NLCs treatment produced more immature SCs with myelin sheath morphology as compared to the nerves treated with FGF9-NLCs (Figure 6C, GAP43 staining). More importantly, nerves tissue treated with FGF9-NLCs showed greater expression of the mature SC marker, myelin basic protein (MBP) and therefore indicated successful re-myelination (Figure 6C, MBP staining). The promotion of regenerated nerve was illustrated by gross images of innervated gastrocnemius muscles (left for injured nerve and right for health leg) and the quantification of relative gastrocnemius muscle weight (RGMW) among different groups (Figure 6D) (p < 0.05). Significant improvement was observed in innervated muscle following treatment with FGF9-NLCs; this was further confirmed by investigating the cross-sectional area of muscle fibers in order to demonstrate successful re-innervation and avoid muscular atrophy (Figure 6D, muscle fiber) (p < 0.05).

**Figure 7:**
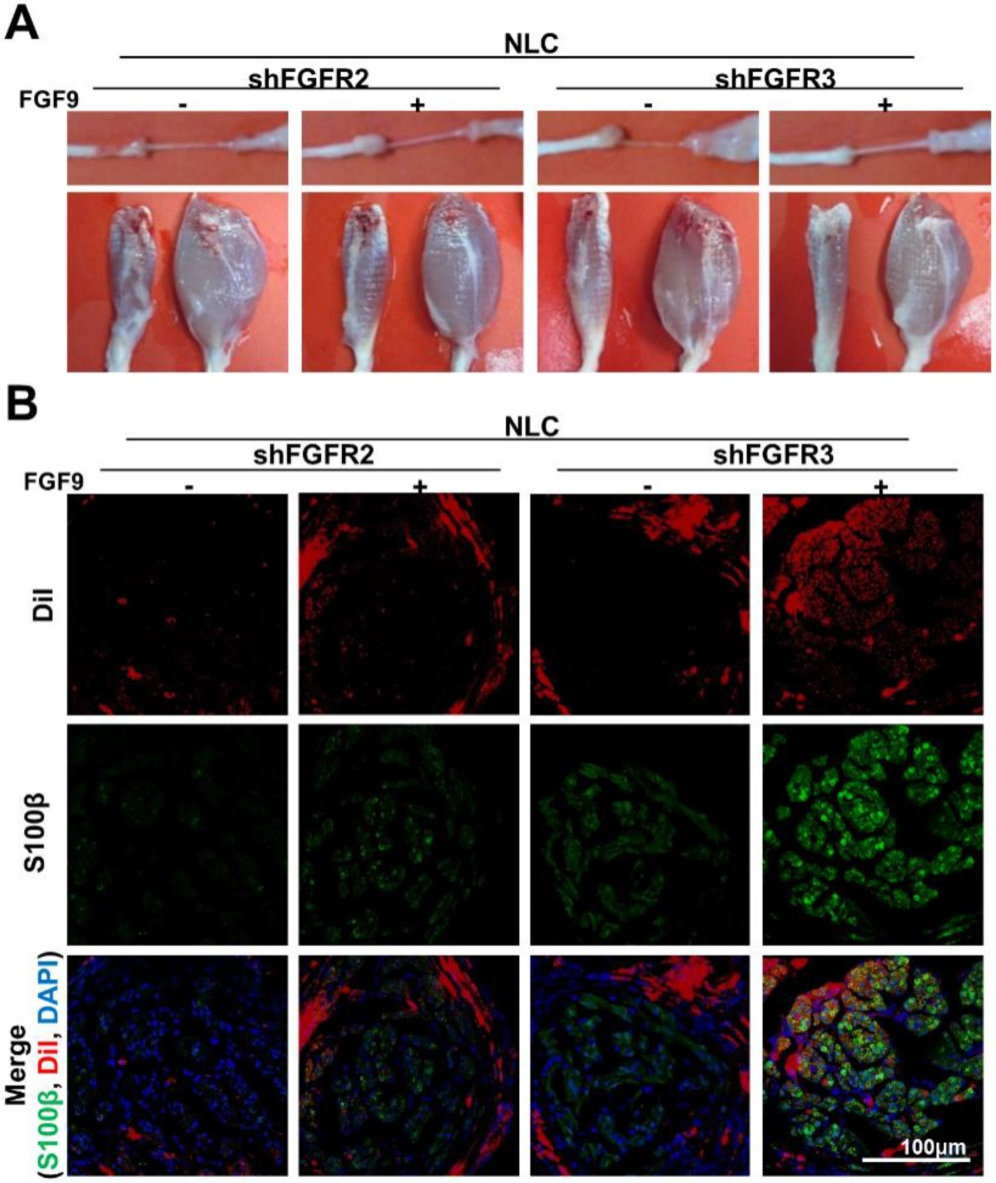
** shFGFR2, but not shFGFR3, abolished the ability of FGF9-NLCs to promote remyelination.** (A) Either NLCs, or FGF9-NLCs with FGFR2 or FGFR3 silencing, were transplanted into the nerve conduit. The gross morphology of the nerve and gastrocnemius muscle suggested that silencing of FGFR2 hindered the beneficial effect of FGF9-NLCs in the promotion of nerve regeneration. (B) IF staining of nerve sections showed that silencing FGFR2, but not FGFR3, in FGF9-NLCs prevented interaction between cells and axons, and thus most of the Dil positive cells were not labeled by S100β staining. Scale bar in fluorescent image: 100 μm.

The benefits of applying an SC lineage using FGF9-NLCs to facilitate nerve regeneration were further investigated by studying the importance of FGFR2-Akt signaling by knocking down FGFR2 or FGFR3 in FGF9-NLCs prior to injection (Figure 7). Silencing FGFR2 in either NLCs or FGF9-NLCs diminished nerve regeneration as illustrated by the gross morphologies of nerve and innervated muscle, but not for cells in which FGFR3 was silenced (Figure 7A). The immunofluorescent staining of S100β showed that silencing of FGFR2 diminished myelin sheath formation, even though the DiI-labeled FGF9-NLCs were still retained in the sectioned tissue (Figure 7B). In contrast, shFGFR3 did not alter the therapeutic effect of FGF9-NLCs. Collectively, this data indicates that FGF9-FGFR2-Akt signaling is essential to derive SCs in NLCs and that fate-committed FGF9-NLCs can contribute to the re-myelination process during the repair of peripheral nerve.

## Discussion

In this study, we illustrate, for the first time, that the supplementation of FGF9 during the induction of NLCs from ASCs can achieve a fate-commitment towards SCs. Moreover, FGFR2 plays a critical role in the differentiation of SCs by activating downstream Akt phosphorylation. The therapeutic effect of ASCs-derived SCs was demonstrated by sciatic nerve transection injury in rats. The microenvironment of stem cells during maintenance or differentiation is highly valued 36, 37. Here, we demonstrate the concept of combining biomaterial (chitosan-coated surface) and biochemical factors (FGF9) to fine tune the neural differentiation microenvironment for ASCs. The autologous cell-based therapy of ASCs for peripheral nerve regeneration was accomplished by inducing Schwann cells via FGF9-NLCs induction to provide cells that can directly participate in re-myelination and functional regeneration. The abundant number of ASCs attracts many studies to use it as delivery platform for CRISPR-based activation of endogenous neurotrophic genes 12 or nanoparticle-engineered the CXCR4-overexpressing cells 38 for potential treatments.

The induction of specific neurons or glial cells from stem cells requires the delivery of combined factors at a precise time and dose in order to mimic developmental cues 19, 39. SCs can be derived by 2-3 weeks induction with MSCs using a growth factor cocktail, including glial growth factors (GGF2), FGF2, platelet-derived growth factor (PDGF) and forskolin 40, 41. The differentiated SCs exhibited a spindle-like morphology and positive markers for GFAP, S100β and p75. By seeding MSCs from a different origin onto a low-attachment surface and by supplementation with EGF and FGF2, cells tend to aggregate into sphere-like structures and express high levels of early neuroectodermal markers, such as otx1, GFAP, Pax6 and Nestin 42-44. Alternative shorter induction protocols have previously been developed by subjecting MSCs to transient hypoxia, followed by neurosphere culture upon low-attachment supplements of EGF and FGF2, and then seeded onto poly-D-lysine/laminin-coated tissue culture plastic and cultured in a gliogenic cocktail to generate SC-like cells 45. These studies shed light on the benefit of sphere culture to facilitate neural differentiation in a relatively short period of time. A hypoxic microenvironment in the culture environment or within the spheres may be critical for enhancing cell survival and initiating the cell fate interconversion of MSCs 46. In addition to the sphere formation, the present finding suggested that supplementation with FGF9 can significantly shift the fate determination of NLCs into S100β-positive SC lineage within 72 hr.

FGF9 has been studied extensively for its role in controlling neurogenesis during development of the CNS, particularly in the differentiation of astrocytes. In the developing brain, FGF9, together with EGFR, acts to promote the astrogenesis rate in the cerebral cortex 47. FGF9 is also highly expressed by neurons, and functions as a communicator between neurons and glial cells to regulate differentiation, migration and myelination 48, 49. However, some studies have shown that FGF9 strongly inhibits the expression of GFAP in NPCs isolated from the subventricular zone of adult mice, and prohibits the differentiation of astrocytes 50. FGF9 also reduces the expression of FGFR2 and myelination proteins in immature oligodendrocytes, such as CNP, PLP and MBP. However, FGF9-FGFR2 signaling promotes the myelination of mature oligodendrocytes 48, 51. The role of FGF9 in regulating the differentiation or maturation of glia cells in the PNS still remains unclear. The effect of direct FGF9 administration was tested in PNS injury 52. FGF9 inhibited the dedifferentiation of SCs and accelerated the accumulation of macrophages during Wallerian degeneration. Mice with Fgf9 conditional knockout in SCs was developed to demonstrate the absence of Fgf9 led to delayed myelination in early development 53. In the developing nerves, Fgf9 knockout mice showed decreased mature SC-related genes and increased the immature SCs genes. FGF9 also induces a pro-inflammatory environment during injury. Here, we showed that FGF9 induced GFAP, S100β, and Nestin expression via the FGFR2 axis without altering the levels of FGFR2 in neurospheres derived from ASCs.

Cells respond differently to FGFs due to differences in cellular content, including divergent FGFR expression profiles in different cells and changes in the binding affinity between FGFs and FGFRs 54. FGF9 has been reported to exhibit the best affinity with FGFR2 and FGFR3 and reduced binding affinity with FGFR1 and FGFR4. In our study, NLCs showed a remarkable increase in FGFR2 and FGFR4 expression, while FGFR3 expression in NLCs was reduced during sphere formation. This considerable change in the profile of FGFRs suggested that the cell characteristics and response to FGFs in NLCs are no longer the same as ASCs. Beside adding FGFs play key roles in neural differentiation of several stem cells 55, 56, our study also demonstrated the knockdown of FGFRs markedly decreased the expression of neural markers, including Nestin, NFH, and GFAP, to highlight the importance of these receptors for specific FGFRs in regulating neural differentiation. Our results also illustrated that FGF9 has the potential to bind to different FGFRs in adherent ASCs and suspended NLCs. The development of prolong FGF9 expressions on modified chitosan surface may provide some convenient device to benefit the stem cell in SC differentiation. Although the FGF9-NLCs showed homogenous expression of GFAP and S100β, it is possible that heterogeneity of gene expression within spheres may still occur in different cells. Since the interaction of FGF9 with different FGFRs may also target different downstream signals and differentiation outcomes, future studies can use quantitative PCR 57 or single cell sequencing 58 to dissect the population of FGF9-NLCs at the single cell level. For example, FGF9 induces FGFR2 signaling to activate the Akt or ERK pathway for downstream signaling and the transcriptional controls of SC lineage differentiation in FGF9-NLCs can therefore be further dissected by sorting with different maturation markers.

In summary, we have provided evidence that FGF9 can induce the fate-determination of SCs from NLCs during sphere formation. The FGF9-FGFR2-Akt pathway plays a major role in mediating SC differentiation. The differentiated FGF9-NLCs might directly participate in myelination to promote the regeneration of injured peripheral nerve. Our results not only provided insight into FGF9-mediated SC differentiation but also indicated an alternative cell source for autologous cell therapy.

## Materials and Methods

### Primary Culture of Adipose-derived Stem Cells and Neurosphere Formation

Human ASCs were isolated from liposuction aspirates obtained from healthy donors with informed consent to protect the client information and patient rights as approved by the Institutional Review Board (IRB) of the National Cheng Kung University Hospital (IRB approval number: B-ER-105-080). The lipoaspirates were washed thoroughly with phosphate buffered saline (PBS), followed by digestion with collagenase (Invitrogen) at 37°C for 30min. The solution was mixed with Dulbecco's modified Eagle's medium (DMEM, Invitrogen) containing 10% fetal bovine serum (FBS, HyClone) to stop collagenase activity and then centrifugation at 1000 rpm for 10min. Stromal cells obtained in the pelleted fraction were then transferred to a culture dish containing DMEM supplemented with 10% FBS and 1% penicillin/streptomycin (Invitrogen) 59. The stem cell characteristics and differentiation properties of ASCs were confirmed and only used within 6 passages 60, 61.

The differentiation of NLCs from ASCs was established as described in previous studies 16, 62. Briefly, 1% w/v chitosan (Sigma-Aldrich) was coated onto tissue culture plates, followed by 1N of NaOH solution and PBS washing. ASCs were seeded onto a chitosan-coated surface at a cell density of 2 × 10^4^ cells/cm^2^, and induced cells were harvested at different time points for analysis. To test the effect of FGF9 in controlling NLCs differentiation, different concentrations (10, 25, 50, 100 ng/mL) of FGF9 (Abcam) were supplemented into the culture medium.

### Determination of Gene and Protein Expression

The fate determination and FGFR profile of NLCs was analyzed by gene and protein expression using various markers of neural lineage, including Nestin, NFH, NeuN, GFAP and S100β. The gene expressions for osteogenesis were tested by Aggrecan, type II collagen (COL2), and SOX9. Gene expression levels were measured using reverse transcription polymerized chain reaction (RT-PCR) by lysing adherent cells or neurospheres with Trizol (Invitrogen) to isolate mRNA which was then reverse transcribed into cDNA using Super Script III (Invitrogen) 15. Taq-polymerase chain reaction (PCR, GeneDirex) was then used to amplify target genes with specific primers (Table 1). The PCR products were measured by gel electrophoresis to confirm the product base pair length and expressions levels after 25-30 cycles. Other immature SCs markers 63, such SOX10 and Oct6, were also observed by using qPCR with the specific primers sequence listed in Table 1.

The protein expression of neural markers, FGFRs, and the downstream molecules involved in various signaling pathways were assessed by western blotting 60. Briefly, the cells and/or neurospheres were lysed with radioimmunoprecipitation assay (RIPA) buffer to extract protein. An equal amount of protein was then separated by sodium dodecyl sulfate polyacrylamide gel electrophoresis (SDS-PAGE) and then transferred onto nitrocellulose membranes (Bio-Rad). The membranes were then hybridized with specific primary antibodies followed by incubation with HRP-conjugated secondary antibodies (Millipore). The specific neural markers used included Nestin (1:1000, MAB5326, Millipore) for neural progenitor/stem cells (NPCs), NFH (1:1000, N0142, Sigma-Aldrich) for mature neurons, GFAP (1:1000, MAB3402, Millipore) for glial cells and S100β (1:500, ab52642, Abcam) for Schwann cells. For each protein, the fold change in expression was determined by the enhanced chemiluminescence system (Millipore) and then quantified by Image J software with normalized to the internal control of β-actin (Image J, NIH). In addition, immunofluorescent staining was also used to confirm protein expression in adherent ASCs and spheroid NLCs.

The intracellular signals during sphere formation were then probed by common Akt and MAPK specific antibodies, including p-Akt (1:1000, #9271, Cell Signaling), Akt (1:500, sc-271149, Santa Cruz), p-ERK (1:1000, #9106, Cell Signaling), ERK (1:500, sc-154, Santa Cruz), p-JNK (1:1000, #9251, Cell Signaling), and JNK (1:500, sc-474, Santa Cruz). Phosphorylation levels were quantified by normalizing the protein expression of phosphorylated antibody to the total one using Image J software (Image J, NIH). Specific FGFR shRNA or phosphorylation inhibitors were used to verify the downstream signaling pathway involved in FGFR-FGF9 signaling. ASCs were transfected with specific shRNA for FGFR1, FGFR2, FGFR3, and FGFR4 (Table 2) by electroporation (NEPA21 Electroporator, Nepagene) using a poring pulse voltage of 175V for 2.5 msec. After electroporation, the transfected ASCs were kept in adherent culture for 24hr for recovery and then seeded onto a chitosan-coated surface for sphere formation. The PI3K/Akt inhibitor LY294006 (10μM, Sigma-Aldrich), and the ERK inhibitor PD98059 (10μM, Sigma-Aldrich), were added into the culture medium during sphere formation to inhibit Akt and ERK phosphorylation, respectively. The inhibitory outcomes were assessed by specific phosphorylation levels using western blotting.

### Rat Sciatic Nerve Injury and Cell Applications

A sciatic nerve transection model was created in 6-week old Sprague Dawley (SD) rats in order to evaluate the therapeutic potential of FGF9 or NLCs, as described in a previous study 16. The experimental procedures and animal care for nerve injury were also reviewed and approved by the IACUC of National Cheng Kung University (IACUC approval number: 105224). In short, a 10-mm gap was created in the sciatic nerve, and the gap was bridged using a chitosan-coated silicon conduit. ASCs (1.2
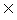
10^6^ cells) were labeled with DiI fluorescent dye (Invitrogen) and then formed neurospheres with or without FGF9 treatment for 3 days. The DiI-labeled NLCs were then dissociated into single cells and applied into the conduit. Six weeks after injury, the nerve tissues were harvested from the proximal, middle (nerve conduit), and distal segments to evaluate regeneration capacity by using histological and immunofluorescent staining. The gastrocnemius muscles were also harvested from both legs and the relative muscle weight (RGMW) was measured by comparing the muscle weight on the injured leg (left) to the intact leg (right) from the same rat to indicate the success of nerve re-innervation and the prevention of muscle atrophy.

### Histological Assessments

The immunofluorescent staining was used in the tissue sectioned that requires multi-color staining. The morphology and composition of tissue will be first checked by Hematoxylin and eosin (H&E) staining. To quantify and verify the myelin sheath structures, at least two different methods for Toludine Blue, semi-thin section, or TEM were used to double confirm the regeneration properties. Briefly, the harvested tissue samples were fixed in 4% paraformaldehyde overnight and embedded either in paraffin for H&E staining (Leica Biosystems) or optimal cutting temperature (OCT, Leica Biosystems) compound for frozen section in immunostaining. To minimize the morphological variances across different regions of regenerating tissue, only the middle position (2.5 mm from center to each end of the nerve in conduit) were sectioned in this study. H&E staining was performed on paraffin-embedded samples to observe the gross morphology of tissue. Immunohistochemistry (IHC) staining was performed to illustrate the expression of specific proteins with cell morphological features, such as glial scar formation by using GFAP (1:500, Millipore), and then using DAB (KPL) reagent to allow color development. Immunofluorescent (IF) staining was used to visualize specific cell types and trace the transplanted cells. For in vitro experiments, the cells were fixed by 4% paraformaldehyde, permeabilized by Triton X-100, blocking by 10% BSA, and then incubated with primary antibody for overnight. For tissue sample processed for IF staining, the harvested nerve was fixed by 4% paraformaldehyde overnight and embedded in OCT for frozen section. The primary antibodies used included NFH (1:200, MAB5448, Millipore), S100β (1:200, ab52642, Abcam), GAP43 (1:200, ab75810, Abcam), MBP (1:50, #78896, Cell Signaling), Thy1 (1:200, ab11575, Abcam), Laminin (1:100, ab11575, Abcam), and p75NTR (1:200, #8238, Cell Signaling). The stained sections were observed with a tissue scanning fluorescent microscope (BX51, Olympus) and confocal microscopy (FV3000, Olympus). Images were acquired from at least three random visual fields and then quantified by Image J software (Image J). To quantify nerve regeneration and re-myelination, an equal number of fibers were randomly selected in each acquired image and the axon diameter within the myelin sheath was measured using Image J. The area of myelin sheath was also calculated by subtracting the area occupied by the axon from the area occupied by the whole nerve fiber. The myelin G-ratio was also calculated after acquired the inner and the outer diameter of the myelin sheath. To quantify the cross-section area of muscle fiber, the muscle fibers were quantified in each randomly acquired image by manually identified the muscle fiber boarder and calculated the area using Image J software (Image J).

### Statistical Analyses

For all experiments, at least 3 independent groups were analyzed and the results were expressed as the mean ± standard deviation. Statistical analysis was performed using one-way analysis of variance (ANOVA) and two-way ANOVA with Tukey's post-hoc test. P-Values of *p* < 0.05 were considered to be statistically significant and were derived using Origin statistic software (version 8.5, OriginLab) and GraphPad Prism (version 8.1).

## Supplementary Material

Supplementary figures.

## Figures and Tables

**Table 1:** Primer pairs for RT-PCR

**Nestin**	Forward	AAGTCTGCGGGACAAGAGAA
	Reverse	TGGTCCTTCTCCACCGTATC
**NeuN**	Forward	GAGGGACGGAAAATTGAGGT
	Reverse	CTGCCGTAACTGTCGCTGTA
**GFAP**	Forward	CTGGAGGTTGAGAGGGACAA
	Reverse	ATACTGCGTGCGGATCTCTT
**S100β**	Forward	ATGTCTGAGCTGGAGAAGGC
	Reverse	TCGTGGCAGGCAGTAGTAAC
**FGFR1**	Forward	ATGGTTGACCGTTCTGGAAG
	Reverse	GGGGTTTGCCTAAGACCAGT
**FGFR2**	Forward	CGCTGGTGAGGATAACAACA
	Reverse	TGACATAGAGAGGCCCATCC
**FGFR3**	Forward	CAAATGGGAGCTGTCTCG
	Reverse	TGCAGGTGTCGAAGGAGTAG
**FGFR4**	Forward	CCTGGTAGAGAACGCTGTGG
	Reverse	GTAGGAGAGGCCGATGGAAT
**Aggrecan**	Forward	GCACTCCCACAGTTGACAGA
	Reverse	AGTCTCCCACTCCAGAAGCA
**COL2**	Forward	CGAGGTGACAAAGGAGAAGC
	Reverse	GGTTGTTCAGCGACTTGAGC
**SOX9**	Forward	AGTTTGACCAATACCTGCCG
	Reverse	AAGGTTGAAGGGGCTGTAGG
**SOX10**	Forward	CAACCACCCCGAAGACAGAG
	Reverse	GGTCCAACTCAGCCACATCA
**OCT6**	Forward	GTTCGCCAAGCAGTTCAAGC
	Reverse	TCCTCCAGCCACTTGTTGAG
**GAPDH**	Forward	CATCAAGAAGGTGGTGAAGC
	Reverse	TGACAAAGTGGTCGTTGAGG

**Table 2:** Sequence of shRNA

**shFGFR1**	CCGGCCCTCCCAGATGTTGGACCAACTCGAGTTGGTCCAACATCTGGGAGGGTTTTTG
**shFGFR2**	CCGGTTAGTTGAGGATACCACATTACTCGAGTAATGTGGTATCCTCAACTAATTTTTG
**shFGFR3**	CCGGCTCGACTACTACAAGAAGACACTCGAGTGTCTTCTTGTAGTAGTCGAGTTTTT
**shFGFR4**	CCGGGCCGACACAAGAACATCATCACTCGAGTGATGATGTTCTTGTGTCGGCTTTTT
