# Supplementary material for: FGF9 induces functional differentiation to Schwann cells from human adipose derived stem cells
Source: Theranostics. 2020 Feb 3;10(6):2817–31. doi: 10.7150/thno.38553 (PMC7052907; doi:10.7150/thno.38553)
Supplement: Supplementary file 1 — Supplementary figures. [file thnov10p2817s1.pdf]

Figure S1

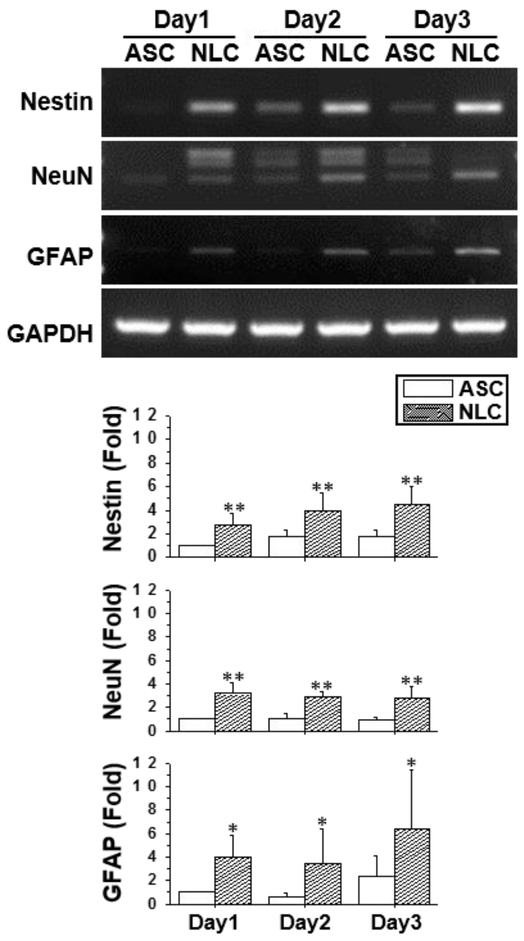

Figure S1. The gene expression of neural markers were observed by RT-PCR on day1 to day3 during the NLCs induction. Data are represented as mean ± SD. N = 3 independent biological repeats. *p < 0.05, **p < 0.01 compared to undifferentiated ASCs.

Figure S2

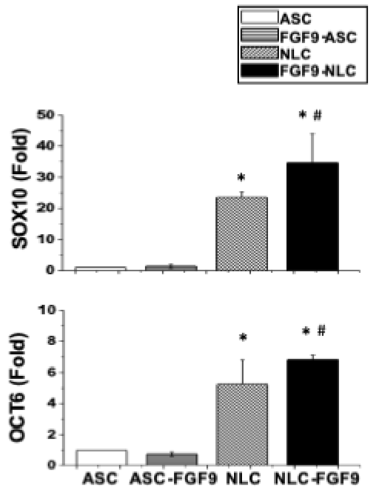

Figure S2. The gene expression of immature SC markers Sox10 and Oct6 were increased in NLCs. The administration of FGF9 further enhanced the expression of Sox10 and Oct6. Data are represented as mean ± SD. N = 3 independent biological repeats. *p < 0.05 compared to undifferentiated ASCs. # p < 0.05 compared to NLCs without FGF9.

Figure S3

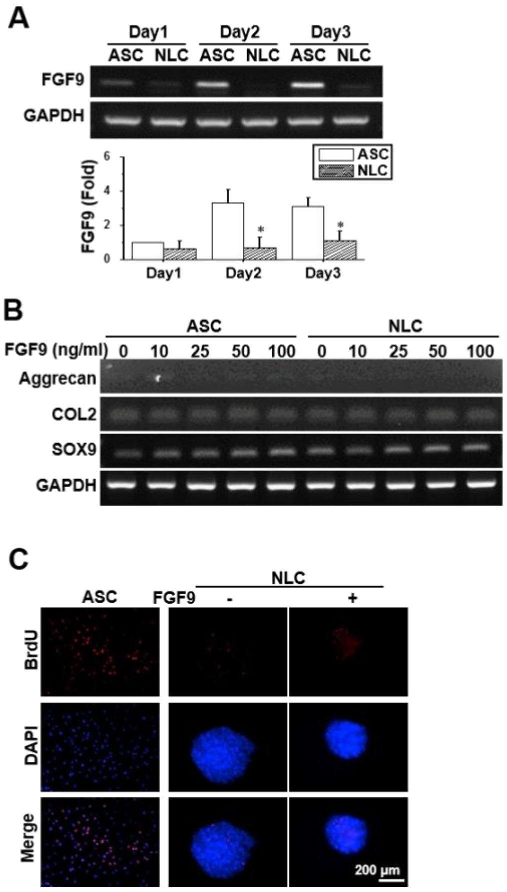

Figure S3. (A)The gene expression of FGF9 were observed by RT-PCR on day1 to day3 during the NLCs induction. (B) The undetectable change of aggrecan, collagen II (COL2), and Sox9 in ASCs and NLCs indicated no chondrogenic differentiation with different dosage of FGF9 treatments. (C) The BrdU was added into the medium during sphere formation to label the proliferating cells (Red color) and demonstrated very few NLCs were positive to BrdU as compared to the adherent ASCs. Furthermore, the addition of FGF9 had no considerable effect on increasing proliferation in NLCs. Data are represented as mean ± SD. N = 3 independent biological repeats. $*p < 0.05$ compared to undifferentiated ASCs.

Figure S4

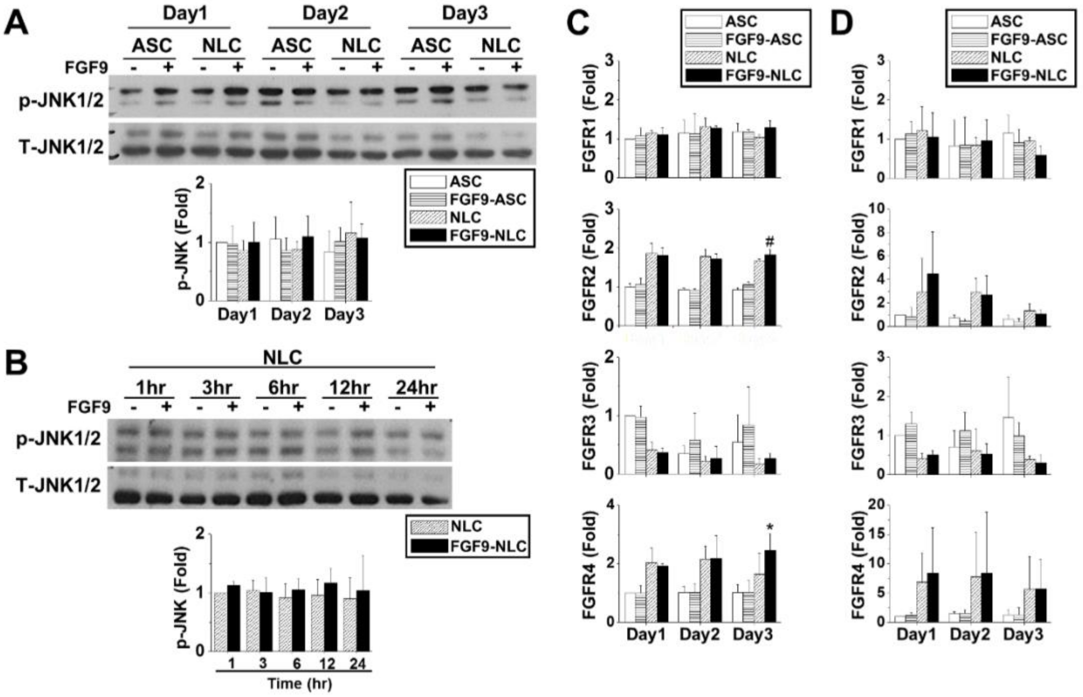

Figure S4. (A) The phosphorylation of JNK was investigated with/without FGF9 treatments between Day1 to Day3 in ASCs and NLCs. (B) The effect of FGF9 on p-JNK were also measured in NLCs at shorter time points within 24 hr. The phosphorylation of JNK was not affected by sphere formation as well as by FGF9 treatment. (C & D) The quantification result of Figure 3E & 3F. Data are represented as mean ± SD. N = 3 independent biological repeats. For C, *p < 0.05 compared to undifferentiated ASCs, # p < 0.05 compared to day1 undifferentiated ASCs.

Figure S5

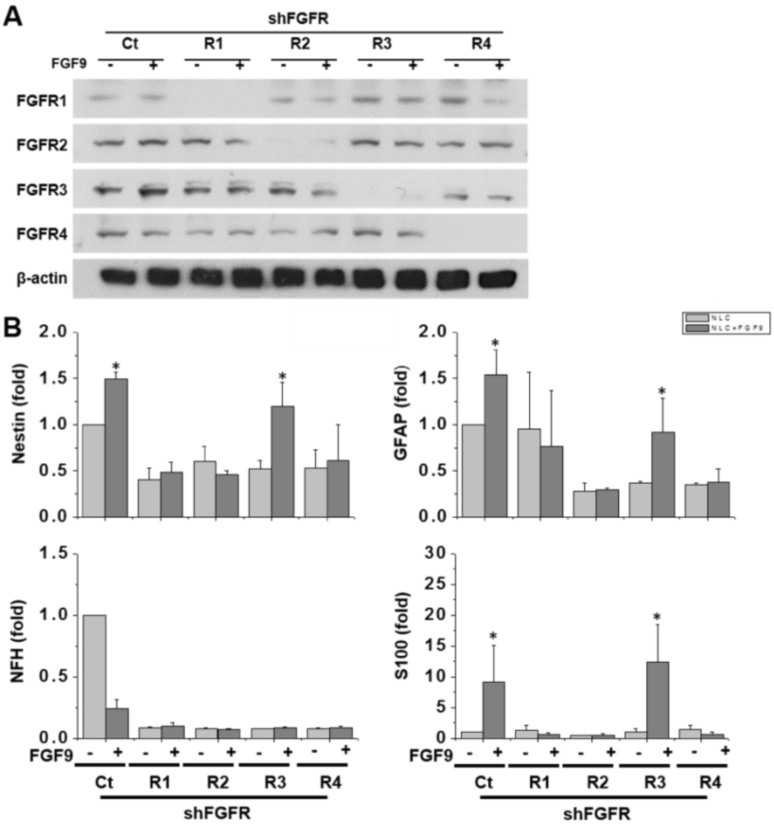

Figure S5. (A) The ASCs were transfected with specific shFGFR1-4 before NLCs induction. The specificity and efficiency of each shFGFR was confirmed by western blotting with specific antibody against FGFR1-4. (B) The quantification result of Figure 4B. Data are represented as mean ± SD. N = 3 independent biological repeats. For B, *p < 0.05 compared to undifferentiated ASCs.

Figure S6

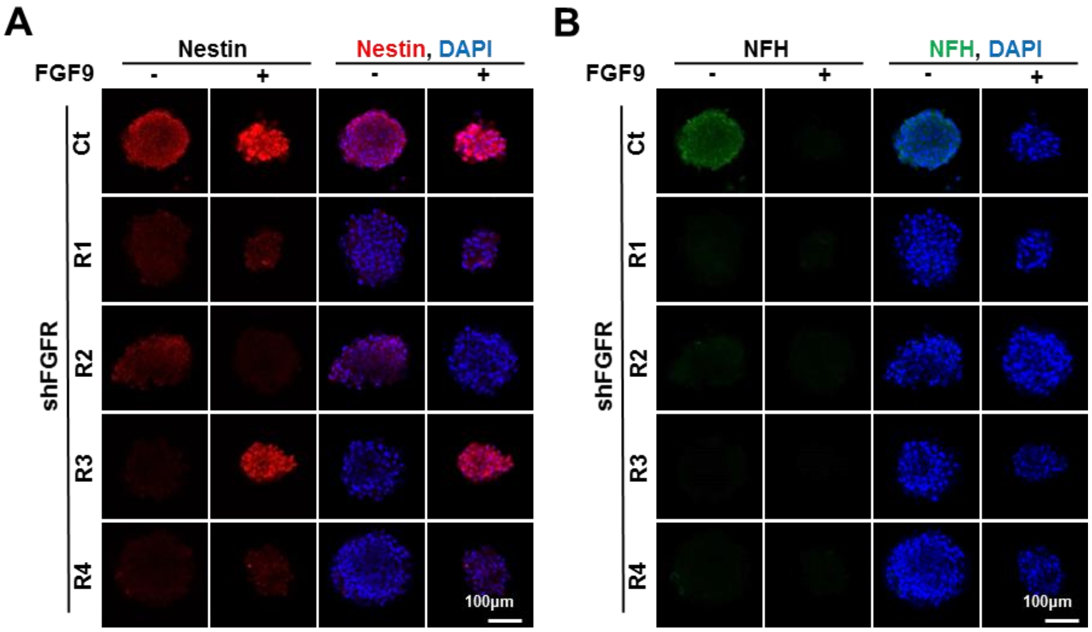

Figure S6. (A) The immunofluorescent staining of Nestin showed the blockage of FGFR2 (shFGFR2 with or without FGF9) inhibited the increase of Nestin expression with FGF9 treatment (Ct with FGF9). Other shFGFR for R1 and R2 also prohibited the Nestin expressions in NLCs, but not shFGFR3. (B) Silencing of any FGFR decreased the NFH expression in NLCs without FGF9 treatment. Scale bar in fluorescent image: 100 µm.

Figure S7

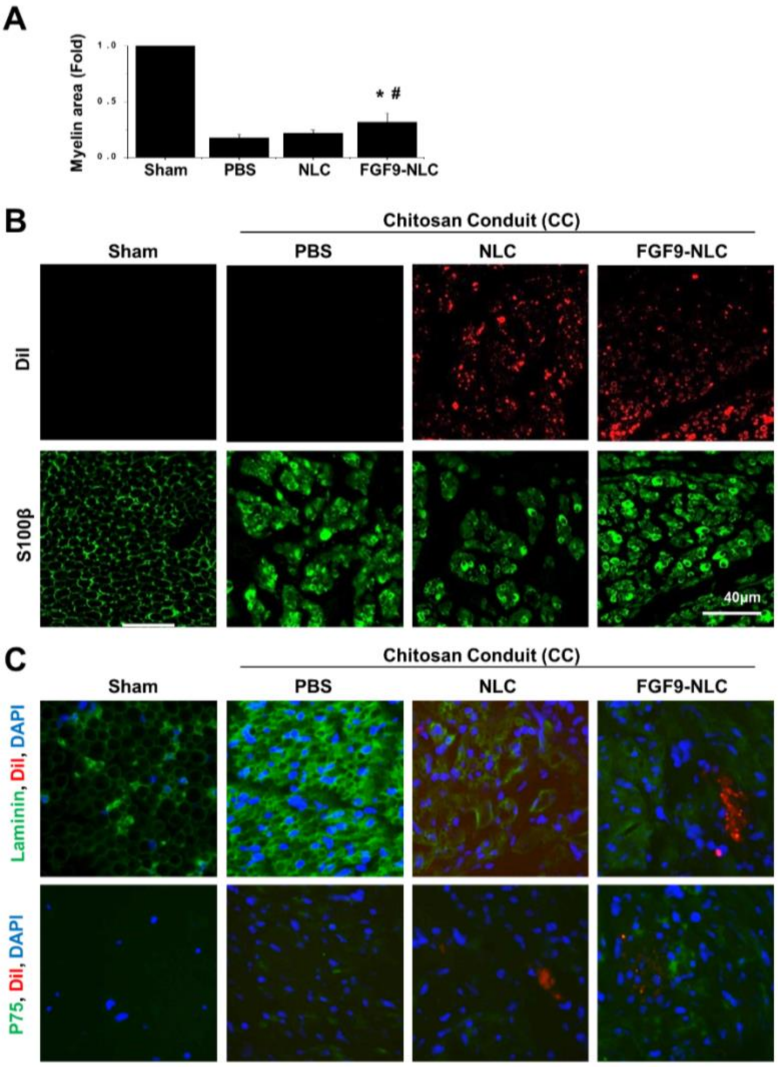

Figure S7. (A) The quantification of myelin sheath area showed improvement in FGF9-NLCs treatment. (B) The immunofluorescent staining of S100β (green) on nerve section. The DiI-labeled cells were tracked by red fluorescent image in the same section. (C) The fibrotic scar occurred by laminin staining in PBS group was decreased in both NLC and FGF9-NLC treatment. Scale bar in fluorescent image: 40 μm. *$p < 0.05$ compared to PBS. #$p < 0.05$ compared to NLC.
